# Development of key interventions and quality indicators for the management of an adult potential donor after brain death: a RAND modified Delphi approach

**DOI:** 10.1186/s12913-018-3386-1

**Published:** 2018-07-24

**Authors:** Pieter Hoste, Eric Hoste, Patrick Ferdinande, Koenraad Vandewoude, Dirk Vogelaers, Ann Van Hecke, Xavier Rogiers, Kristof Eeckloo, Kris Vanhaecht, on behalf of the Donation after Brain Death Study Group, Liesbet De Bus, Dirk Rijckaert, Joris Vermassen, Patrick Biston, Charlotte Castelain, Els Colla, Kirsten Colpaert, Jacques Creteur, Johan De Blanger, Annick De Weerdt, Bruno Desschans, Patrick Evrard, Denis Glorieux, Philippe Hantson, Anne Joosten, Josée Monard, Baudewijn Oosterlynck, Alain Roman, Riet Minnekeer, Gerda Van Beeumen, Sophie Van Cromphaut

**Affiliations:** 1 Department of General Internal Medicine, Ghent University Hospital, Corneel Heymanslaan 10, 9000 Ghent, Belgium; 2 Faculty of Medicine and Health Sciences, Ghent University, Corneel Heymanslaan 10, 9000 Ghent, Belgium; 3 Department of Internal Medicine, Ghent University, Corneel Heymanslaan 10, 9000 Ghent, Belgium; 4 Department of Intensive Care, General Hospital Sint-Lucas, Groenebriel 1, 9000 Ghent, Belgium; 5 Department of Intensive Care Medicine, Ghent University Hospital, Corneel Heymanslaan 10, 9000 Ghent, Belgium; 6 Research Foundation - Flanders (FWO), Egmontstraat 5, 1000 Brussels, Belgium; 7 Surgical and Transplantation ICU, University Hospitals Leuven, Herestraat 49, 3000 Leuven, Belgium; 8 University Centre for Nursing and Midwifery, Ghent University, Corneel Heymanslaan 10, 9000 Ghent, Belgium; 9 Department of Public Health, Ghent University, Corneel Heymanslaan 10, 9000 Ghent, Belgium; 10 Nursing Department, Ghent University Hospital, Corneel Heymanslaan 10, 9000 Ghent, Belgium; 11 Department of Transplant Surgery, Ghent University Hospital, Corneel Heymanslaan 10, 9000 Ghent, Belgium; 12 Leuven Institute for Healthcare Policy, Department of Public Health and Primary Care, KU Leuven - University of Leuven, Kapucijnenvoer 35, 3000 Leuven, Belgium; 13 Department of Quality Management, University Hospitals Leuven, Herestraat 49, 3000 Leuven, Belgium; 14 European Pathway Association, Kapucijnenvoer 35, 3000 Leuven, Belgium

**Keywords:** Delphi technique, Key interventions, Quality indicators, Critical care, Deceased donation, Donation after brain death

## Abstract

**Background:**

A substantial degree of variability in practices exists amongst donor hospitals regarding the donor detection, determination of brain death, application of donor management techniques or achievement of donor management goals. A possible strategy to standardize the donation process and to optimize outcomes could lie in the implementation of a care pathway. The aim of the study was to identify and select a set of relevant key interventions and quality indicators in order to develop a specific care pathway for donation after brain death and to rigorously evaluate its impact.

**Methods:**

A RAND modified three-round Delphi approach was used to build consensus within a single country about potential key interventions and quality indicators identified in existing guidelines, review articles, process flow diagrams and the results of the Organ Donation European Quality System (ODEQUS) project. Comments and additional key interventions and quality indicators, identified in the first round, were evaluated in the following rounds and a subsequent physical meeting. The study was conducted over a 4-month time period in 2016.

**Results:**

A multidisciplinary panel of 18 Belgian experts with different relevant backgrounds completed the three Delphi rounds. Out of a total of 80 key interventions assessed throughout the Delphi process, 65 were considered to contribute to the quality of care for the management of a potential donor after brain death; 11 out of 12 quality indicators were validated for relevance and feasibility. Detection of all potential donors after brain death in the intensive care unit and documentation of cause of no donation were rated as the most important quality indicators.

**Conclusions:**

Using a RAND modified Delphi approach, consensus was reached for a set of 65 key interventions and 11 quality indicators for the management of a potential donor after brain death. This set is considered to be applicable in quality improvement programs for the care of potential donors after brain death, while taking into account each country’s legislation and regulations regarding organ donation and transplantation.

**Electronic supplementary material:**

The online version of this article (10.1186/s12913-018-3386-1) contains supplementary material, which is available to authorized users.

## Background

Organ transplantation has proven to be lifesaving and to have improved the quality of life of numerous patients since the first successful kidney transplant in 1954. As the standard treatment for end-stage organ failure, organ transplantation is currently performed in 112 countries worldwide. In 2015, more than 143,000 patients across the 47 member states of the Council of Europe were on waiting lists for a heart, lung, kidney, liver, pancreas or intestinal transplant. Unfortunately, on average 18 of them died every day because of lack of timely organ availability [1]. The majority of transplant procedures rely on organs from donors after brain death (DBD). DBDs are more likely to donate multiple transplantable organs. The maintenance of perfusion and oxygenation in DBDs creates optimal conditions for successful organ transplantation.

In order to cope with these transplant needs, the field of organ donation and transplantation has been forced to evolve rapidly. Various health care services are required in this complex care process and therefore an effective organization and coordination of all involved health care professionals is essential. Nowadays, in many European Union member states, donor coordinators have been appointed in hospitals with an intensive care unit (ICU), where organ retrieval from deceased donors can be considered. Donor coordinators have clearly defined responsibilities in establishing, managing and reviewing the deceased donation processes in their hospital [2]. To support this, guidelines for the management of potential donors can provide donor coordinators with recommendations based on the best available evidence. However, in spite of efforts to develop standardized guidelines, there remains a large degree of variability in practices amongst hospitals regarding the determination of brain death, application of donor management techniques or achievement of donor management goals [3–7]. These may potentially contribute to under-recruitment of potential organ donors.

A possible strategy to standardize the donation process and to optimize outcomes could lie in the implementation of a validated care pathway. Care pathways are defined by the European Pathway Association as ‘a complex intervention for the mutual decision making and organization of care processes for a well-defined group of patients during a well-defined period’ [8]. They support the translation of clinical guidelines into local protocols and introduction into clinical practice [9]. Care pathways are used worldwide for a variety of patient groups to reduce undesired variability and standardize care based on the latest evidence [10]. They have also been developed for donation after brain death, such as the pathways of the United Network for Organ Sharing, National Institute for Health and Clinical Excellence or National Health Service Blood and Transplant [11–13]. However, a recent systematic review on the effects of existing care pathways for donation after brain death revealed that only one study effectively evaluated the impact of such a care pathway [14].

Typical active ingredients of a care pathway include the promotion of interdisciplinary teamwork, the integration of a set of evidence-based key interventions (KI), and the active follow-up of care processes by a set of quality indicators (QI) to verify compliance to KIs [15]. KIs are those which are required to guarantee high quality care, and hence in this setting will have a significant impact on patient, donor family, recipient or graft outcomes.

The present study therefore aims at selecting a set of KIs to be included in a care pathway for donation after brain death as well as a set of QIs that are relevant to assess the quality of care for potential DBDs and the impact of such a care pathway.

## Methods

### Study design

To develop a set of relevant KIs and QIs, a RAND modified Delphi technique [16] was used with a predefined number of rounds to stop the Delphi process and a threshold value for consensus [17]. After selection of an extensive set of KIs and QIs from the literature and composition of a multidisciplinary expert panel, three anonymous questionnaire rounds and one physical meeting were performed to achieve panel consensus about the relevance of the proposed KIs and relevance and feasibility of the proposed QIs. Questionnaires were conducted through LimeSurvey®, an open-source software tool to conduct online surveys [18]. E-mail reminders were sent at 2 weeks following the initial email of each round. The consensus procedure took place between March and June 2016.

### Composition of expert panel

The objective was to generate a multidisciplinary Delphi panel of physicians and nurses involved in the donation process after brain death in Belgium in order to guarantee relevance for clinical practice and generalizability of results [17, 19]. The main eligibility criteria consisted of a longstanding experience in the field of organ donation, preferably for a minimum of 10 years, and a minimum of 3 organ donors throughout 2015 in the donor hospital, in which the expert was professionally active.

All Belgian donor coordinators (*n* = 196), the board members of the Belgian Society of Intensive Care Medicine (*n* = 8), and the members of the Transplant Coordinators Section (*n* = 28) and the Belgian Organ Procurement Committee (*n* = 19) of the Belgian Transplantation Society were invited to join this study by an information letter (Additional file 1) sent by e-mail by the first author (PH), describing the criteria required to be involved in this Delphi panel.

### Selection of key interventions and quality indicators

The selection of KIs and QIs consisted of 8 steps: (1) Delphi questionnaire preparation with extraction of KIs and QIs, (2) first Delphi round, (3) data analysis of the first round, (4) second Delphi round, (5) data analysis of the second round, (6) third Delphi round, (7) data analysis of the third round, and (8) physical consensus meeting.

#### Step 1: Delphi questionnaire preparation with extraction of key interventions and quality indicators

To develop a Delphi questionnaire including all possible relevant and feasible KIs and QIs, an extensive literature review was conducted by the first author (PH). For the review of guidelines on the management of a potential DBD, the following resources were explored: (I) Websites of national European transplantation organizations or societies: Agence de la biomédecine, British Transplantation Society, Deutsche Stiftung Organtransplantation, Nederlandse Transplantatie Stichting, NHS Blood and Transplant, and Organización Nacional de Trasplantes; (II) Websites of European transplantation or intensive care medicine organizations or societies: European Directorate for the Quality of Medicines and HealthCare, European Society of Intensive Care Medicine, European Society of Organ Transplantation, Eurotransplant, and Scandiatransplant; (III) Websites of international transplantation societies: International Liver Transplantation Society, International Transplant Nurses Society, The International Society for Heart & Lung Transplantation, and The Transplantation Society; (IV) Public resources for evidence-based clinical practice guidelines: Guidelines International Network, National Guideline Clearinghouse, National Institute for Health and Clinical Excellence, and Scottish Intercollegiate Guidelines Network; (V) Process flow diagrams based on evidence-based medicine: Map of Medicine and National Institute for Health and Clinical Excellence; and (VI) Electronic databases: MEDLINE, CINAHL and EMBASE.

For the first 5 resources, the following search terms were used: ‘organ donation’ and ‘brain death’. For the electronic database MEDLINE, the Medical Subject Headings (MeSH) terms ‘brain death’, ‘donor selection’, ‘tissue and organ harvesting’, ‘tissue and organ procurement’ or ‘tissue donors’ were used in combination with ‘guideline’ or ‘practice guideline’, both as publication type. The strategy was translated for the other databases. Search limit parameters included: (I) published between 2009 and 2015, and (II) written in English, Dutch or French.

Only few of these guidelines included KIs for donor management [20–22]. Therefore, an additional search was performed in the electronic databases, MEDLINE, CINAHL, EMBASE and The Cochrane Library, to include recent review articles, using the search term ‘donor management’. In addition to the QIs listed in the guidelines and review articles, the QIs identified in the organ donation process of the Organ Donation European Quality System (ODEQUS) project were also analyzed. These were developed by a consortium involving associated and collaborating partners from 16 European countries [23].

A two-phase screening evaluation of publications from these resources was applied. In the first phase, publications were appraised for relevance based on appropriateness of the title and abstract. If relevance was unclear, or if the abstract was unavailable, the full text of these publications was assessed. In the second phase, the full text of the selected guidelines or process flow diagrams were reviewed. Following inclusion criteria were applied: (I) descriptions of KIs or QIs regarding an adult patient with a devastating brain injury or lesion with evolution to imminent brain death until post procurement, and (II) underpinning by in-text references of evidence to support their practice. The guidelines selected after full text review were appraised using the validated AGREE II-Global Rating Scale (AGREE II-GRS) quality assurance tool for clinical practice guidelines. This instrument consists of 4 items assessing the quality of guideline reporting. Each item is scored on a seven-point scale [24]. Guideline quality was independently rated by three reviewers (PH, KV and PF). A consensus meeting was held between these reviewers to determine the mean score of the overall guideline quality. Disagreements between reviewers during quality rating were resolved through discussion until consensus was reached. Only clinical practice guidelines with a mean score of 5–7 points on the overall guideline quality were included.

After the extensive literature review, potential KIs and QIs were selected by PH, EH and PF. These KIs and QIs were integrated in an internet-based Delphi questionnaire, consisting of three main parts: demographic questions (name and type of hospital or organization, number of intensive care beds, number of organ donors, professional group, function, years of experience in organ donation, age and gender), KIs and QIs. The demographic questions are included in the Additional file 1. The provisional Delphi questionnaire was pretested by three intensivists, who were not eligible to participate in the expert panel.

#### Step 2: Delphi round 1

During the first round, the participants received an e-mail with a link to the internet-based Delphi questionnaire. In addition to the demographic information, experts were asked to provide comments on the listed KIs and QIs or add new ones.

#### Step 3: Data analysis of Delphi round 1

Based on the comments in Delphi round 1, adjustments with regard to the description of the KIs and QIs were made and KIs or QIs were deleted. Newly identified KIs or QIs suggested by the expert panel were included in the questionnaire.

#### Step 4: Delphi round 2

In preparation for the second round, the participants received feedback of all the first-round panel members’ comments, deleted KIs and QIs, and the additionally proposed KIs and QIs. In the first part of the second Delphi round, experts were asked to rate on a 9-point Likert rating scale (score 1 indicating “strongly disagree”; score 9 “strongly agree”), to what extent each KI would contribute to the quality of care for the management of a potential donor (or the donor family, recipient or graft) and similarly to which extent each QI could be considered relevant and/or feasible to be implemented. The KIs & QIs of the Delphi round 2 are presented in the Additional file 1.

#### Step 5: Data analysis of Delphi round 2

The results of the second round were analyzed using predefined consensus criteria based on a systematic review about the use and reporting of the Delphi method for selecting health care QIs [17]. A KI was considered valid if it had a median score of 7 or more with 75% or more of the ratings in the highest tertile (Likert score: 7–9). A QI was accepted with agreement if the attribute relevance had a median score of 7 or more with 75% or more of the ratings in the highest tertile (Likert score: 7–9) and the attribute feasibility had a median score of 7 or more.

#### Step 6: Delphi round 3

In round 3, feedback on the quantitative panel results was provided to all members of the panel, presented by the following summary statistics: central tendencies (median, minimum, maximum, and mode), frequency of ratings in each tertile Likert category (1–3, 4–6, and 7–9), rating of contribution (ratio of “sum of ratings on the intervention given by participants” to “sum of ratings on the intervention if all respondents rated the interventions as ‘strongly agree’”), and the respondent’s own responses. Using this information, respondents were asked to re-rate the KIs and QIs in case they would like to change their previous answers.

#### Step 7: Data analysis of Delphi round 3

The same predefined consensus criteria as in step 5 were applied to the analysis of the results of the third Delphi round. If participants of round 2 did not respond in round 3, their answers of round 2 were considered as final.

#### Step 8: Physical consensus meeting

A face-to-face consensus meeting (June 2016) was organized to discuss and re-rate the KIs and QIs without consensus after the third round [17]. The nominal group technique was used as consensus method [25]. One author (DV) moderated this meeting in order to contain the influence of dominant personalities. Another author (PH) presented the available literature concerning the ‘no consensus’ KIs and QIs. Subsequently, the experts had the possibility to discuss the literature, followed by the opportunity for re-rating previous individual scores using the same Likert rating scale.

## Results

### Delphi panel participants’ characteristics

A total number of 20 eligible experts agreed to participate in this study. The expert panel had an average of 18-year experience in the field of organ donation (Table 1 for more detailed characteristics of the expert panel). In round 1, 18 of 20 invited experts completed the questionnaire. All 18 participants completed the three Delphi rounds. The physical meeting was attended by 9 experts.

**Table 1:** Characteristics of the Delphi panel (*n* = 18)

Characteristics	*n* (%)
Gender	
Male	9 (50)
Female	9 (50)
Age (years)	
30–49	7 (39)
50–69	11 (61)
Professional group	
Medical doctor	11 (61)
Nurse	6 (33)
Other	1 (6)
Functions	
Intensive care medicine	11 (33)
Anesthesiology	2 (6)
Intensive care nursing	4 (12)
Donor coordination	13 (39)
Transplant coordination	3 (9)
Years of experience	
5–9	2 (11)
10–19	7 (39)
20–29	9 (50)
Number of organ donors after brain death and circulatory death in 2015	
3–5	4 (22)
6–9	5 (28)
10–25	9 (50)
Type of institution	
Academic hospital	12 (67)
Non-academic community hospital	6 (33)

### Development of Delphi questionnaire

**Fig. 1:**
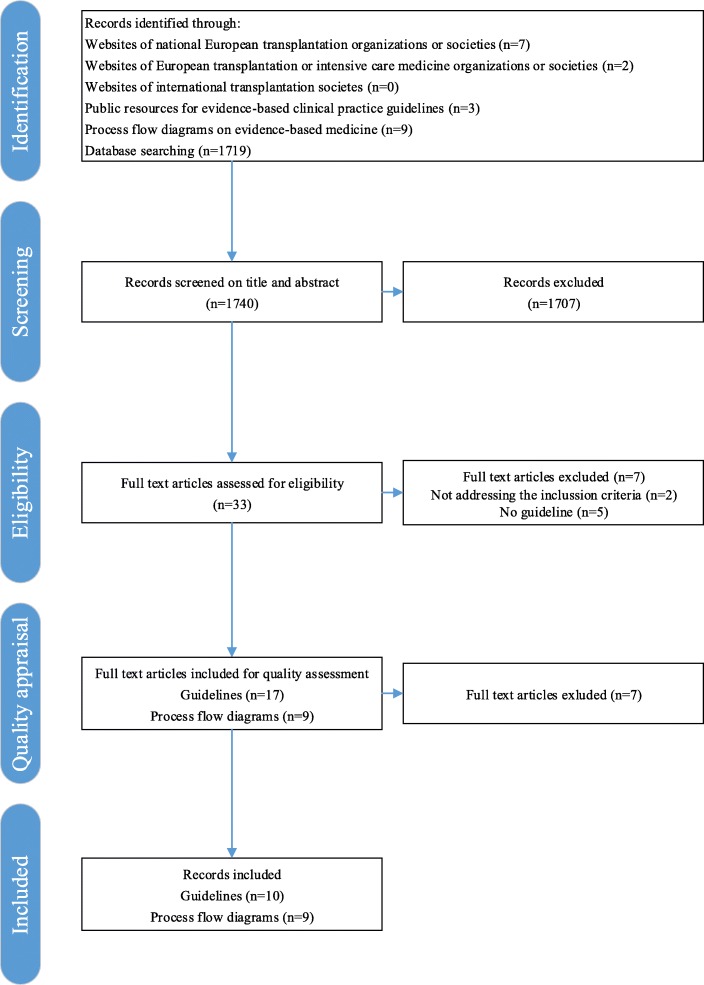
Selection of guidelines and process flow diagrams

The literature research initially revealed 12 guidelines, 9 process flow diagrams, and 1719 digital records from the electronic medical databases. After screening and assessment for eligibility and quality appraisal of full-texts, 10 guidelines [20–22, 26–32] and 9 process flow diagrams [33–41] were included (Fig. 1). In addition, several review articles [42–49] and the results of the ODEQUS project [23] were also included.

Based on the review of the literature, 77 potential KIs and 12 QIs were selected by PH, EH and PF. The KIs were distributed into 10 domains: (I) detection outside the ICU and communication to the ICU (*n* = 1); (II) detection inside the ICU and notification to a transplant center (*n* = 12); (III) donor evaluation and characterization (*n* = 15); donor management: (IV) general care (*n* = 7), (V) monitoring (*n* = 20), (VI) cardiovascular management (*n* = 5), (VII) respiratory management (*n* = 6), (VIII) renal and electrolyte management (*n* = 5), (IX) hormone substitution (*n* = 3); and (X) post procurement care (*n* = 3). The QIs were distributed into 3 domains: (I) structure (*n* = 5), (II) process (*n* = 5), and (III) outcome indicators (*n* = 2) respectively.

### Results of the key interventions

Based on the comments in Delphi round 1, some adjustments with regard to the description of some of the 77 KIs were made and 2 KIs were deleted: ‘*request to a transplant center to perform a liver biopsy in case of hepatic steatosis and ship it to a transplant center for evaluation by a pathologist*’ (donor evaluation and characterization) and ‘*central venous pressure monitoring, which is used as a dynamic measure to assess volume status or fluid responsiveness*’ (donor management: monitoring). There were 5 newly identified KIs suggested by the expert panel, presented in Table 2 and Additional file 2. These additional interventions were situated within the topics: ‘donor evaluation and characterization’, ‘donor management: cardiovascular management and hormone substitution’, and ‘post procurement’.

**Table 2:** Results of the 65 key interventions for which consensus was reached by the overall panel after the third Delphi round

	Based on literature (L) or expert panel (E)	Median	Tertile 7–9 (%)	Tertile 7–9 (n)	Rating of contribution*
Detection outside the ICU & communication to the ICU					
Detection of a patient with a devastating brain injury or lesion with evolution to imminent brain death (for example intracranial hemorrhage, trauma, cerebral ischemia etc.) on a unit outside the ICU (for example emergency services, stroke units, etc.) and early communication of the presence of this patient to the ICU physician (and referral to the ICU).	L	8	89%	16	87%
Detection inside the ICU & notification to a transplant center					
Detection of a potential donor after brain death inside the ICU. Detection should be based on defined clinical triggers in patients who have had a devastating brain injury or lesion, while recognizing that clinical situations vary ˗ A Glasgow Coma Scale score of 4 or less that is not explained by sedation and ˗ The absence of one or more cranial nerve reflexes Unless there is a clear reason why the above clinical triggers are not met and/or a decision has been made to perform brainstem death tests, whichever is the earlier.	L	9	100%	18	94%
Notification of the donor coordinator at the time these criteria are met.	L	9	94%	17	91%
Assessment of the prerequisites prior to the clinical evaluation of brain death: ˗ Coma, irreversible, and cause known. ˗ Neuroimaging compatible with coma. ˗ Central nervous system depressant drug effect absent (if indicated, toxicology screen; if barbiturates given, serum level < 10 μg/mL). ˗ No evidence of residual paralytics (electrical stimulation if paralytics used). ˗ Absence of severe acid-base, electrolyte, and endocrine abnormality. ˗ Normothermia or mild hypothermia (core temperature > 36 °C). ˗ Systolic blood pressure > 100 mmHg. Vasopressors may be required. ˗ No spontaneous respiration.	L	8	89%	16	83%
Approaching the family: ˗ Delivering bad news about the hopeless, medical situation. ˗ Support of the family (physician, nurse, social assistant, psychologist, pastoral service…).	L	9	100%	18	93%
Notification of the potential donor after brain death by an ICUphysician to a transplant center: ˗ Briefing: name, date of birth, diagnosis & therapy, short medical and behavioral history, etc. ˗ Check the medical contra-indications for organ and tissue donation on file with the transplant center. ˗ Is there a registration in the National Register, checked by the transplant center?	L	9	89%	16	91%
Determination of brain death.	L	9	100%	18	95%
Legal declaration of death: registration of time of death and the way in which it is determined on a dated and signed official report.	L	9	89%	16	93%
Notification of legal authorities if the cause of death is unknown or suspicious.	L	9	89%	16	90%
Informing the family about the diagnosis of brain death.	L	9	100%	18	98%
Informing the family about the outcome of the National Register and the possibility of organ and tissue donation, preferably in a separated conversation after family understand and accept the diagnosis of brain death.	L	9	94%	17	94%
Give clear, unambiguous information about the next main steps about the donation process to the relatives.	L	9	100%	18	96%
Feedback about the approach of the family and legal authorities (if the cause of death is unknown or suspicious) and discussion about the necessary investigations for donor evaluation and characterization to a transplant center.	L	9	89%	16	90%
Donor evaluation and characterization					
Interviewing family and/or other relevant sources (e.g. life partner, cohabitant, caretaker, friend or primary care physician) to obtain the medical and behavioral history of the potential donor which might affect the suitability of the organs for transplantation and imply the risk of disease transmission.	L	8	89%	16	89%
Reviewing medical charts to obtain the medical and behavioral history of the potential donor which might affect the suitability of the organs for transplantation and imply the risk of disease transmission.	L	9	89%	16	93%
Clinical examination of the potential donor.	L	9	89%	16	91%
Collect a blood sample and ship it to a transplant center for appropriate blood tests.	L	9	100%	18	93%
Discuss with a transplant center, the necessity to examine a blood sample for the determination of ABO, rhesus blood group or additional laboratory tests.	L	9	83%	15	90%
Collect a urine sample (if not shipped to a transplant center) for measurement of sediment, protein & glucose.	L	9	83%	15	87%
Perform a chest X-ray, mandatory for each potential donor and to allow evaluation of a potential lung and/or heart donor.	L	9	89%	16	90%
Discuss with a transplant center, the necessity to perform a bronchoscopy by an experienced physician to allow evaluation of a potential lung donor together with a bilateral bronchoalveolar lavage to collect samples for microbiological tests and to clear mucous plugs or blood clots that may contribute to impaired oxygenation.	L	8	78%	14	81%
Perform an arterial blood gas to allow evaluation of a potential lung donor.	L	9	83%	15	88%
Discuss with a transplant center, the necessity to perform an arterial blood gas for a potential lung donor after 10 min ventilation with FiO_2_ 100% & 5 cm H_2_O PEEP.	L	9	83%	15	89%
Perform a 12 lead ECG to allow evaluation of a potential heart donor.	L	9	89%	16	90%
Discuss with a transplant center, the necessity to perform a cardiac ultrasound by an experienced physician to allow evaluation of a potential heart donor.	L	9	89%	16	89%
Discuss with a transplant center, the necessity to perform, if possible, a coronary angiography if cardiac ultrasound is acceptable but other comorbidities are present.	E	8	89%	16	86%
Discuss with a transplant center, the necessity to perform an abdominal ultrasound (or CT scan) to allow evaluation of a potential liver, pancreas and/or kidney donor.	L	8	94%	17	88%
Collect the minimum data, as requested by the transplant center for the characterization of organs and donor, on a donor information form and send it together with the results of the investigations to a transplant center.	L	9	100%	18	93%
Donor management: general care					
Provide at least an arterial line and a central venous line, if not present.	L	8	83%	15	86%
Continue appropriate antibiotic therapy and other life supporting pharmacotherapy, only if indicated.	L	8	94%	17	90%
Use warming mattress, blankets or warmed intravenous fluids if needed, to prophylactically prevent hypothermia.	L	8	78%	14	84%
Reduce vasopressors (if possible) while maintaining hemodynamic stability.	L	9	100%	18	92%
Donor management: monitoring					
Monitor the core body temperature. Target temperature: between 35 and 37 °C.	L	8	100%	18	91%
ECG monitoring of heart rate. Target heart rate between 60 and 100 beats per minute.	L	8	78%	14	83%
Repeat a 12-lead ECG for a potential heart donor if there are subsequent changes in monitored complexes.	L	8	83%	15	87%
Invasive arterial pressure monitoring. Target mean arterial pressure: ≥ 60 mmHg.	L	9	94%	17	91%
Ensuring a recent chest X-ray examination for a potential lung and/or heart donor is available.	L	9	89%	16	90%
Monitoring of ventilator parameters.	L	9	94%	17	91%
Peripheral oxygen saturation monitoring (SaO_2_). Target SaO_2_: > 95%.	L	9	83%	15	91%
Perform a blood gas analysis on a regular basis. Target pH: 7.3–7.5. Target arterial oxygen tension (PaO_2_): 80–100 mmHg. Target arterial carbon dioxide tension (PaCO_2_): 35–45 mmHg.	L	8	89%	16	88%
Send a bronchial secretion sample for microscopy and culture if secretions are present.	L	8	89%	16	89%
Perform a bronchoscopy for diagnosis or therapy if clinically indicated.	L	8	83%	15	88%
Estimate the effective intravascular volume and overall fluid status by chart review and clinical examination.	L	8	78%	14	81%
Monitor hourly urine output, particularly looking for any suggestion of the onset of diabetes insipidus (polyuria). Target urine output: 0.5–3 mL/kg/h.	L	8	89%	16	90%
Measure blood electrolytes on a regular basis.Target serum sodium: ≤ 155 mEq/L.	L	8	89%	16	87%
Measure routine full blood counts to examine the need for transfusion of red blood cells if clinically indicated. Target hemoglobin: > 7 g/dL.	L	8	78%	14	81%
Donor management: cardiovascular management (hypotension)					
Use isotonic crystalloids for intravascular volume replacement and use blood products and colloids (albumin) for specific circumstances.	L	8	94%	17	90%
Ensuring an appropriate prescription of vasoactive drugs when correction of the volume deficit fails to achieve the threshold hemodynamic goals.	L	9	100%	18	92%
Donor management: cardiovascular management (bradycardia)					
Treat bradycardia causing hemodynamic instability, with a short acting β-adrenergic agonist (epinephrine/dopamine/dobutamine/isoprenaline) or occasionally transvenous pacing. Don’t use atropine because bradycardia are the consequence of high-level vagal stimulation and exhibit a high degree of resistance to atropine.	L	7	83%	15	81%
Donor management: cardiovascular management (tachycardia)					
Treat tachycardia by following the established advanced cardiopulmonary life support guidelines.	E	8	89%	16	87%
Donor management: respiratory management					
Ensuring a lung protective ventilation is installed: ˗ Minimum FiO_2_ to obtain a PO_2_ between 80 and 100 mmHg ˗ Tidal volume (Vt): 6–8 mL/kg (ideal body weight) ˗ Plateau pressure: < 30 cm H_2_O ˗ PEEP (Positive End Expiratory Pressure): 8–10 cm H_2_O	L	8	89%	16	85%
Maintain 30–45° head of bed elevation to avoid aspiration.	L	8	89%	16	89%
Perform recruitment maneuvers and repeat when indicated.	L	8	83%	15	85%
Apply a prescription of oral hygiene every 6 h.	L	7	89%	16	84%
Donor management: renal and electrolyte management (oliguria < 0.5 mL/kg/h)					
Treat hypovolemia, hypotension and cardiac dysfunction and consider diuretic only if needed.	L	9	100%	18	93%
Donor management: renal and electrolyte management (polyuria > 3 mL/kg/h)					
Review the medical history, urinary and blood sample to exclude secondary polyuria: osmotic (Mannitol, hyperglycemia), induced (diuretic) or adapted (fluid overload).	L	8	100%	18	90%
Confirm diabetes insipidus: urine specific gravity below 1.005 g/mL or trend towards hypernatremia/hyperosmolarity.	L	8	94%	17	87%
Treat diabetes insipidus with sufficient fluid volume replacement to compensate polyuria and anti-diuretic hormone replacement. ˗ Fluid volume replacement with monitoring of electrolytes and blood glucose levels. ˗ Anti-diuretic hormone replacement with desmopressin as a first line medication.	L	8	100%	18	93%
Donor management: renal and electrolyte management (electrolyte disturbances)					
Treat electrolyte disturbances.	L	9	100%	18	93%
Donor management: hormone substitution					
Ensuring an appropriate prescription of insulin if treating hyperglycemia to achieve a target glucose level of 180 mg/dL or less.	L	8	83%	15	87%
Post procurement care					
Detection, registration and reporting of serious adverse events to the transplant center.	L	9	100%	18	94%
Debriefing by the donor coordinator and/or transplant coordinator about the results of the transplantation (anonymous) to the relatives, health care professionals and primary care physician.	L	9	94%	17	93%
Offering, if necessary, support to the relatives, for example by a feedback conversation after a couple of weeks or information about associations for relatives.	E	9	94%	17	93%
Debriefing with the involved health care professionals and transplant coordinator.	E	9	89%	16	90%
Ensuring the hospitalization invoice of the patient is excluded of any medical, pharmaceutical or hospital costs after the determination of brain death and legal declaration of death.	L	9	94%	17	94%

*rating of contribution = ratio of “sum of ratings on the intervention given by participants” to “sum of ratings on the intervention if all respondents rated the interventions as ‘strongly agree’”

In the second and third round, the experts could rate the now 80 KIs. The full Delphi panel of 18 experts reached consensus for 65 of the 80 KIs after the third round (data given in Table 2 with their respective Likert ratings). These interventions were considered to contribute to the quality of care for the management of a potential donor (or the donor family, recipient or graft). Because not all the experts could attend the physical meeting after round 3, the results about these 65 KIs with Likert weighted consensus were considered as the final results of this Delphi survey.

The 15 KIs without consensus after the third round are displayed in the Additional file 2. In the physical meeting, after discussion of the literature, 9 experts reached consensus about 4 out of the remaining 15 KIs without consensus after the third round: (I) *Continue an appropriate prescription of deep venous thrombosis prophylaxis: low molecular weight heparin* (donor management: general care); (II) *Periodically re-assess cuff pressure to check if there is no cuff leak and if cuff pressure is between 20 and 30 cm H*_*2*_*O to avoid aspiration*; (III) *Ensuring coagulation screening or thromboelastography to target therapy if there is a clinically relevant bleeding*; and (IV) *Monitoring of glycemic status to target blood glucose ≤ 180 mg/dL* (donor management: monitoring). The main reasons for not selecting certain KIs after the third round and physical meeting, as described by the experts, were low level of evidence, the prior inclusion in standard ICU care, conflicting evidence, or rather qualification as an additional intervention rather than a KI.

### Results of the quality indicators

The expert panel did not suggest new QIs or adjustments to the 12 QIs in the first Delphi round. The full Delphi panel of 18 experts reached consensus for 11 of 12 QIs (4 structure, 5 process and 2 outcome indicators) after the third round. In parallel with the KIs, the results about these 11 QIs with Likert weighted consensus were considered as the final results of the Delphi survey (Table 3).

**Table 3:** Results of the 11 quality indicators for which consensus was reached by the overall panel after the third Delphi round

	Attribute	Median	Tertile 7–9 (%)	Tertile 7–9 (n)
Structure indicators				
1. Existence of donation process procedures.	Relevance	9	89%	16
*Formula: existence of procedures for all relevant steps of the donation process?*	Feasibility	9	83%	15
2. Existence of a proactive donor detection protocol.	Relevance	9	89%	16
*Formula: existence of a donor detection protocol?*	Feasibility	8	72%	13
3. Documentation of key interventions of the donation process.	Relevance	8	89%	16
*Formula: existence of a documentation form with all relevant key interventions of the donation process?*	Feasibility	8	83%	15
4. Seminars on organ donation.	Relevance	8	83%	15
*Formula: number of organ donation seminars organized last year?*	Feasibility	8	78%	14
Process indicators				
5. Detection of all potential donors after brain death in the ICU.	Relevance	9	94%	17
*Formula: number of potential donors after brain death in the ICU who are referred to the donor coordinator / number of potential donors after brain death in the ICU.*	Feasibility	8	83%	15
6. Evaluation of donors after brain death.	Relevance	9	89%	16
*Formula: number of patients declared brain death in the ICU who have been evaluated as donors in consult with a transplant center / number of patients declared brain death in the ICU.*	Feasibility	8	78%	14
7. Donor management goals.	Relevance	8	83%	15
*Formula: number of actual donors after brain death in the ICU meeting 5 of the 7 donor management goals prior to organ recovery (mean arterial pressure: 60–110 mmHg, number of vasopressors ≤ 1, arterial blood gas pH: 7.3–7.5, serum sodium: 135–155 mEq/L, blood glucose: ≤ 180 mg/dL, urine output: ≥ 0.5 mL/kg/h over 4 h, core body temperature: 35–37 °C) / number of actual donors after brain death in the ICU.*	Feasibility	8	72%	13
8. Documentation of cause of no donation.	Relevance	9	94%	17
*Formula: number of failed potential donors in which the cause of no donation is properly documented / number of failed potential donors.*	Feasibility	8	83%	15
9. Documentation of evaluation of potential donors.	Relevance	8	83%	15
*Formula: number of donors correctly evaluated / number of donors evaluated.*	Feasibility	8	67%	12
Outcome indicators				
10. Family objection to organ donation.	Relevance	9	89%	16
Formula: number of objections (number of potential donor after brain death cases with family objection to organ donation) / number of families interviewed* (number of potential donor after brain death cases in which family members are informed about the possibility of organ donation). *exclusion of donor cases where the patient’s wishes are known (formal or informal).	Feasibility	8	78%	14
11. Conversion rate in donors after brain death.	Relevance	9	78%	14
*Formula: number of actual donors after brain death / number of eligible donors after brain death.*	Feasibility	9	78%	14

The QI without consensus after the third round is included in Additional file 2 and was not withheld in the physical meeting.

## Discussion

To our knowledge, this is the first report on the selection of a set of KIs that can be used for the clinical content of a care pathway for donation after brain death. A set of 65 KIs was developed as relevant to quality of care. These interventions cover the complete organ donation pathway, including donor detection, brain death determination, family approach, donor evaluation and characterization, donor management, and the post procurement phase. Furthermore, to assess the quality of care for potential DBDs and the impact of this care pathway, a set of 11 QIs was validated for the attributes relevance and feasibility. To include recent data of studies, a continuous monitoring and updating process of this set of KIs and QIs and the resulting donor pathway is obviously needed.

While several guidelines, review articles, and process flow diagrams for the management of a potential donor have been published, there remains a lack of high quality evidence to guide clinical practice. The recommendations are largely based on physiological rationale on the one hand and, consensus statements that overwhelmingly comprised observational studies and retrospective case series on the other hand. This represents low-quality evidence, with a lack of randomized controlled trials [42, 46]. Remarkably however, only 15 of the 80 KIs after the third Delphi round were considered as not valid nor relevant by the expert panel, so consensus was reached for most interventions. This implies that the KIs selected out of the literature are reasonably well in agreement with the opinions of our expert panel, representing a “mainstream” of expert opinion.

The Delphi procedure is an accepted methodology for the selection of KIs and QIs in health care. This systematic approach is recommended in research areas hampered by limited evidence to guide clinical practice and disagreement between experts on its interpretation. This method combines evidence-based practice with expert opinion by using a multidisciplinary panel. A large group of experts across diverse locations and areas of expertise can be included anonymously, thus avoiding domination of the consensus process by one or a few experts. This group facilitation technique is designed to transform individual opinions of experts into group consensus. It includes a series of questionnaires or rounds to gather information and achieve consensus [17, 19, 25].

In this Delphi study, outcomes such as patient and graft survival, graft function, or acute rejection are not included [29]. These are valuable variables but are likely dependent on a number of factors that are not related to the donation procedure (e.g. recipient characteristics, organ procurement, and preservation), and thereby provide less information to guide quality improvements at a donor hospital. Beside QIs related to organ donation, a set of transplant QIs can also be identified. Accountability of the transplant centers on these transplant QIs, will not only stimulate the donor hospitals towards more active engagements in the field but also increase more transparency to the general public [50].

This study was restricted to the phase of KIs and QIs selection. In a next step, further research should explore which KIs (I) are effectively implemented in practice (adherence), and (II) could be improved. These interventions can then be used as a standard to evaluate the quality of existing DBD care and in quality improvement programs. Research should also determine the effect of these interventions on a set of QIs in order to substantiate progress. To this purpose, the three dimensions of structure, process and outcome indicators can be used to assess quality of care [25]. QIs rated as most important were (I) detection of all potential DBDs in the ICU and (II) documentation of cause of not proceeding to donation in potential donors. Reliability and feasibility in practice of this indicator set needs to be tested in both low- and high-volume donor hospitals. With these indicators, donor coordinators could evaluate the quality of the organ donation process at the hospital level.

Our study has several strengths. We used the systematic RAND modified Delphi method, a common and validated technique in which scientific evidence is combined with expert opinion. Our procedure is consistent with the guideline of Boulkedid et al. for using and reporting this consensus technique, in which the median number of panel members was 17 [17]. Our panel was multidisciplinary, with 18 experts covering 5 different functions: intensive care medicine, anesthesiology, intensive care nursing, donor coordination, and transplant coordination. All involved stakeholders were presented. All the experts completed the three Delphi rounds, which implies that we had a low non-response bias, increasing the validity of the results. These are highly relevant and applicable for clinical teams managing potential DBDs in different health systems, while taking into account each country’s legislation and regulations regarding organ donation and transplantation. For being universally accepted, these KIs and QIs need to be tested in an international setting.

However, this study has also some limitations. It is uncertain whether the experts who participated are a true representation of the potentially available experts with preferably a minimum of 10 years’ experience and a minimum of 3 organ donors in 2015. On average, only 32% (*n* = 31) of the Belgian acute care hospitals (*n* = 98) had more than 3 donors in 2012/2013, therefore the majority of the informed donor coordinators did not meet the criteria to participate in this study [50]. A second limitation of this study is the national setting in which these KIs and QIs were selected. However, international literature was reviewed and the QIs development of the ODEQUS project was performed by a multidisciplinary panel, in which several members have international experience and expertise on the topic. Another potential limitation is the attendance of the physical meeting by only 9 experts because of logistic reasons. However, in this meeting only the KIs and QIs without consensus after round 3 were re-rated by the experts present and the results of this meeting were not included in the final results of the Delphi survey. Finally, only literature published in English, Dutch or French was included in this study, which may include language bias for example to Spanish or German literature.

## Conclusions

Using a RAND modified Delphi approach, consensus was reached for a set of 65 KIs for the management of potential DBDs. To assess quality of care for potential DBDs and the impact of this care pathway, 11 QIs were validated for the attributes relevance and feasibility. These KIs are to be considered as a first description of a standard bundle of care for potential DBDs, while the QIs identified can be incorporated into specific quality improvement programs.

## Additional files

Additional file 1: Questionnaire. (DOC 677 kb)

Additional file 2: Additional results of the third Delphi round and the physical meeting. (DOC 113 kb)

## Abbreviations

AGREE: Appraisal of Guidelines for Research & Evaluation
DBD: Donor after Brain Death
ICU: Intensive Care Unit
KI: Key Intervention
MeSH: Medical Subject Headings
ODEQUS: Organ Donation European Quality System
QI: Quality Indicator
