# Supplementary material for: Development of key interventions and quality indicators for the management of an adult potential donor after brain death: a RAND modified Delphi approach
Source: BMC Health Serv Res. 2018 Jul 24;18:580. doi: 10.1186/s12913-018-3386-1 (PMC6056930; doi:10.1186/s12913-018-3386-1)
Supplement: Supplementary file 1 — Questionnaire. (DOC 677 kb) [file 12913_2018_3386_MOESM1_ESM.doc]

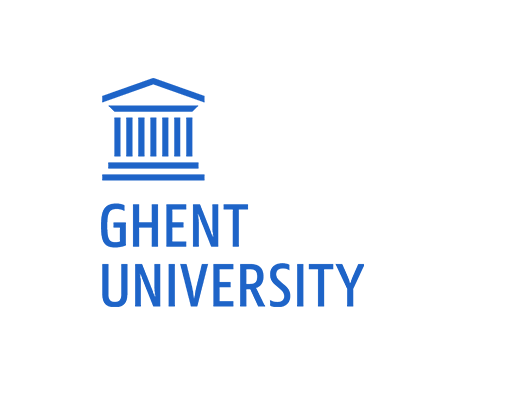


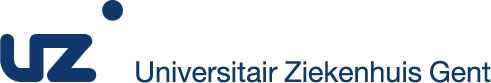


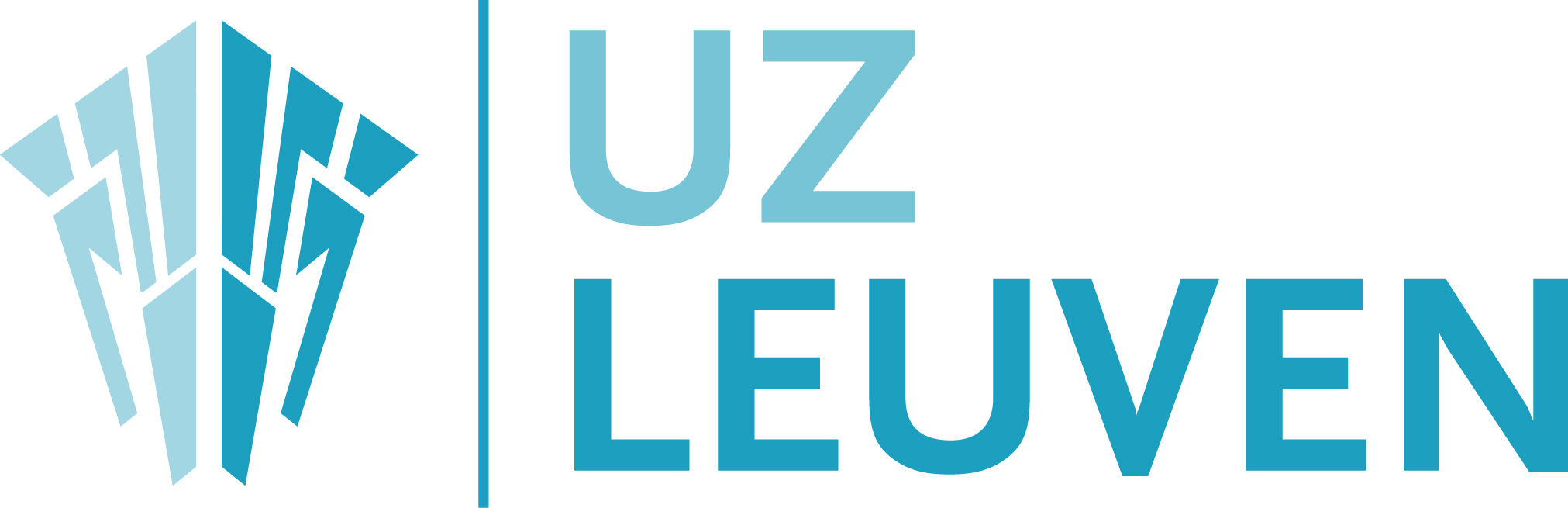


**DONATION AFTER BRAIN DEATH**

**RAND MODIFIED DELPHI METHOD**

Address of correspondence:

[**piehoste.hoste@ugent.be**](mailto:piehoste.hoste@ugent.be)

Pieter Hoste, RN, MSc^1, 2, 3, 4^, Patrick Ferdinande, MD, PhD^5^, Kris Vanhaecht, RN, PhD^6, 7, 8^, Xavier Rogiers, MD, PhD^2, 9^, Kristof Eeckloo, LLM, PhD^2^, Eric Hoste, MD, PhD^2^, ^4^, ^10, 11^, Dirk Vogelaers, MD, PhD^1, 2, 3^, Ann Van Hecke, RN, PhD^2, 12, 13, 14^ & Koenraad Vandewoude, MD, PhD^2, 3^

^1^ Department of General Internal Medicine, Ghent University Hospital, Ghent, Belgium; ^2^ Faculty of Medicine and Health Sciences, Ghent University, Ghent, Belgium; ^3^ Department of Internal Medicine, Ghent University, Ghent, Belgium ^4^ Department of Intensive Care, General Hospital Sint-Lucas, Ghent, Belgium; ^5^ Surgical and Transplantation ICU, University Hospitals Leuven, Leuven, Belgium; ^6^ Leuven Institute for Healthcare Policy, Department of Public Health and Primary Care, KU Leuven - University of Leuven, Leuven, Belgium; ^7^ Department of Quality Management, University Hospitals Leuven, Leuven, Belgium; ^8^ European Pathway Association, Kapucijnenvoer, Leuven, Belgium; ^9^ Department of Transplant Surgery, Ghent University Hospital, Ghent, Belgium; ^10^ Department of Intensive Care Medicine, Ghent University Hospital, Ghent, Belgium; ^11^ Research Foundation - Flanders (FWO), Egmontstraat, Brussels, Belgium; ^12^ University Centre for Nursing and Midwifery, Ghent University, Ghent, Belgium; ^13^ Department of Public Health, Ghent University, Ghent, Belgium; ^14^ Nursing Department, Ghent University Hospital, Ghent, Belgium.

**INFORMATION LETTER**

Care pathways, also known as clinical pathways or critical pathways, are used worldwide for a variety of patient groups to reduce undesired variability and standardize care based on the latest evidence. Nonetheless very few methodologically robust prospective studies have been performed and published on the impact of pathways on quality and efficiency of care. Care pathways have also been developed for donation after brain death in order to optimize the donation process. But only the study of Rosendale et al. (2002) has evaluated the impact of such a care pathway. The goal of this study is to select a set of key interventions to be included in a care pathway for donation after brain death as well as a set of quality indicators that are relevant to assess the quality of care for potential donors after brain death (see glossary) and the impact of this care pathway.

For this purpose, we wish to consult a multidisciplinary Delphi panel of physicians and nurses in Belgium in order to guarantee relevance for clinical practice and generalizability of results. We are looking for experts meeting the following requirements: (1) Involvement in the donation process after brain death; (2) Relevant experience (preferably for a minimum of 10 years) in the field of organ donation; (3) A minimum of 3 organ donors throughout 2015 if the expert is working in a donor hospital; (4) Motivation to complete the Delphi task for the whole process to ensure consensus on this set of key interventions and quality indicators.

If you are able and willing to be involved in this Delphi panel, you can send an email to [**piehoste.hoste@ugent.be**](mailto:piehoste.hoste@ugent.be). An electronic questionnaire will be sent to you. Completion of the questionnaire for each round will take about 45 minutes. After three anonymous rounds (for more details see further on), it may be necessary to organize a physical meeting with the expert panel to discuss points of view. Your judgments and opinions will remain strictly confidential.

The questionnaire is built up in three parts:

*Part I: demographic information (only in round 1)*

In this part, we would like you to fill in some general information to describe our expert panel.

*Part II: selection of key interventions*

Based on guidelines [1-10], process flow diagrams [11-19], and review articles [20-27], a list of key interventions starting from an adult patient with a devastating brain injury or lesion with evolution to imminent brain death until post procurement are defined. Key interventions are those which are required to guarantee high quality care, and hence in this setting will have a significant impact on patient, donor family, recipient or graft outcomes. In a first round you can comment on the listed key interventions or add new ones. In a second round, we would like you to indicate on a 9-point rating scale, if the key interventions will contribute to the quality of care for the management of a potential donor (or the donor family, recipient or graft). In addition, in a third round you will have the possibility to adjust your answers based on the group responses. The aim is to determine the level of consensus about the key interventions which should be included in a care pathway for donation after brain death.

*Part III: selection of quality indicators*

Based on the results of the ODEQUS project [28] and the guidelines [1-10], process flow diagrams [11-19], and review articles [20-27], a selection of quality indicators for assessing the quality of care are defined. In a first round you can comment on the listed quality indicators or add new ones. In a second round, we would like you to evaluate this set of indicators on relevance and feasibility on a 9-point rating scale. In addition, in a third round you will have the possibility to adjust your answers based on the group responses. The aim is to determine the level of consensus about the indicators which should be used to study the quality of care and the impact of a care pathway for donation after brain death.

We would like to thank you in advance for your effort in helping us with this project. Please do not hesitate to contact us if you have any questions.

**QUESTIONNAIRE PART I:**

**DEMOGRAPHIC INFORMATION**

1. **Name:** ……………………………………………
2. **Name of your hospital / organization:** ……………………………………………
3. **Type of hospital:**

Academic hospital

Non-academic community hospital

Other: …………………………………

1. **Number of intensive care beds in your hospital / organization:** … **beds**
2. **Number of organ donors after brain death and circulatory death in your hospital / organization:**
   - 2013: …………..
   - 2014: …………..
   - 2015: …………..
3. **Professional group:**

Medical doctor

Nurse

Other: ……………………………………

1. **Function (multiple answers possible):**

Intensivist

Anesthesiologist

Urgentist/emergency physician

ICU nurse

Donor coordinator

Transplant coordinator

Procurement surgeon

Researcher

Government

Other (please specify):

1. **Years of experience in organ donation:** … **years**
2. **Age:**

20-29  30-39  40-49  50-59  60-69  > 70

1. **Sex:**

Male  Female

**QUESTIONNAIRE PART II:**

**KEY INTERVENTIONS FOR DONATION AFTER BRAIN DEATH (DELPHI ROUND 2)**

**First round**: please comment on the listed key interventions if they are not well formulated for you or add new ones.

*For example: key intervention X*

*Comments on this key intervention: …………………………………………………………..………….....*

*Reference(s): ………….………………………………………………………………………………………..*

**Second & third round**: please indicate on a 9-point rating scale, if the key intervention will contribute to the quality of care for the management of the potential donor (or the donor family, recipient or graft), with 1 indicating “strongly disagree” and 9 “strongly agree”.

*For example: key intervention X*

| Strongly disagree |  |  |  | Undecided |  |  |  | Strongly agree |
| --- | --- | --- | --- | --- | --- | --- | --- | --- |
| 1 | 2 | 3 | 4 | 5 | 6 | 7 | 8 | 9 |
|  |  |  |  |  |  |  |  |  |

**Definition of consensus** **after the third round**

A key intervention will be considered valid if it has a median score of 7 or more with 75% of more of the ratings in the highest tertile (Likert score: 7-9).

| **Detection outside the ICU & communication to the ICU** |
| --- |
| 1. Detection of a patient with a devastating brain injury or lesion with evolution to imminent brain death (for example intracranial hemorrhage, trauma, cerebral ischemia etc.) on a unit outside the ICU (for example emergency services, stroke units, etc.) and early communication of the presence of this patient to the ICU physician (and referral to the ICU). |
| **Detection inside the ICU & notification to a transplant center** |
| 1. Detection of a potential donor after brain death inside the ICU.   Detection should be based on defined clinical triggers in patients who have had a devastating brain injury or lesion, while recognizing that clinical situations vary   - A Glasgow Coma Scale score of 4 or less that is not explained by sedation and - The absence of one or more cranial nerve reflexes   Unless there is a clear reason why the above clinical triggers are not met and/or a decision has been made to perform brainstem death tests, whichever is the earlier. |
| 1. Notification of the donor coordinator at the time these criteria* are met.   *A Glasgow Coma Scale (GCS) score of 4 or less that is not explained by sedation and the absence of one or more cranial nerve reflexes. |
| 1. Assessment of the prerequisites prior to the clinical evaluation of brain death:  - Coma, irreversible, and cause known. - Neuroimaging compatible with coma. - Central nervous system depressant drug effect absent (if indicated, toxicology screen; if barbiturates given, serum level < 10 µg/mL). - No evidence of residual paralytics (electrical stimulation if paralytics used). - Absence of severe acid-base, electrolyte, and endocrine abnormality. - Normothermia or mild hypothermia (core temperature > 36°C). - Systolic blood pressure > 100 mm Hg. Vasopressors may be required. - No spontaneous respiration. |
| 1. Approaching the family:  - Delivering bad news about the hopeless, medical situation. - Support of the family (physician, nurse, social assistant, psychologist, pastoral service…). |
| 1. Notification of the potential donor after brain death by an ICU physician to a transplant center:  - Briefing: name, date of birth, diagnosis & therapy, short medical and behavioral history, etc. - Check the medical contra-indications for organ and tissue donation on file with the transplant center. - Is there a registration in the National Register, checked by the transplant center? |
| 1. Determination of brain death. |
| 1. Legal declaration of death: registration of time of death and the way in which it is determined on a dated and signed official report. |
| 1. Notification of legal authorities if the cause of death is unknown or suspicious. |
| 1. Informing the family about the diagnosis of brain death. |
| 1. Informing the family about the outcome of the National Register and the possibility of organ and tissue donation, preferably in a separated conversation after family understand and accept the diagnosis of brain death. |
| 1. Give clear, unambiguous information about the next main steps about the donation process to the relatives. |
| 1. Feedback about the approach of the family and legal authorities (if the cause of death is unknown or suspicious) and discussion about the necessary investigations for donor evaluation and characterization to a transplant center. |
| **Donor evaluation and characterization** |
| 1. Interviewing family and/or other relevant sources (e.g. life partner, cohabitant, caretaker, friend or primary care physician) to obtain the medical and behavioral history of the potential donor which might affect the suitability of the organs for transplantation and imply the risk of disease transmission. |
| 1. Reviewing medical charts to obtain the medical and behavioral history of the potential donor which might affect the suitability of the organs for transplantation and imply the risk of disease transmission. |
| 1. Clinical examination of the potential donor. |
| 1. Collect a blood sample and ship it to a transplant center for appropriate blood tests. |
| 1. Discuss with a transplant center, the necessity to examine a blood sample for the determination of ABO, rhesus blood group or additional laboratory tests. |
| 1. Collect a urine sample (if not shipped to a transplant center) for measurement of sediment, protein & glucose. |
| 1. Perform a chest X-ray, mandatory for each potential donor and to allow evaluation of a potential lung and/or heart donor. |
| 1. Discuss with a transplant center, the necessity to perform a bronchoscopy by an experienced physician to allow evaluation of a potential lung donor together with a bilateral bronchoalveolar lavage to collect samples for microbiological tests and to clear mucous plugs or blood clots that may contribute to impaired oxygenation. |
| 1. Perform an arterial blood gas to allow evaluation of a potential lung donor. |
| 1. Discuss with a transplant center, the necessity to perform an arterial blood gas for a potential lung donor after 10 minutes ventilation with FiO_2_ 100% & 5 cm H_2_O PEEP. |
| 1. Perform a 12 lead ECG to allow evaluation of a potential heart donor. |
| 1. Discuss with a transplant center, the necessity to perform a cardiac ultrasound by an experienced physician to allow evaluation of a potential heart donor. |
| 1. Discuss with a transplant center, the necessity to perform, if possible, a coronary angiography if cardiac ultrasound is acceptable but other comorbidities are present. |
| 1. Discuss with a transplant center, the necessity to perform an abdominal ultrasound (or CT scan) to allow evaluation of a potential liver, pancreas and/or kidney donor. |
| 1. Collect the minimum data, as requested by the transplant center for the characterization of organs and donor, on a donor information form and send it together with the results of the investigations to a transplant center. |
| **Donor management: general care** |
| 1. Provide at least an arterial line and a central venous line, if not present. |
| 1. Continue enteral feeding until otherwise instructed by the transplant center. |
| 1. Continue appropriate antibiotic therapy and other life supporting pharmacotherapy, only if indicated. |
| 1. Continue an appropriate prescription of deep venous thrombosis prophylaxis (low molecular weight heparin). |
| 1. Ensuring a prescription of low-dose dopamine with a dose of (and not exceeding) 4 µg/kg/min until the aortic clamping and halve the dosage or terminate the infusion earlier when circulatory adverse effects occurred in association with the dopamine infusion, such as tachycardia (> 120 beats per min) or a marked increase in blood pressure (MAP > 110 mm Hg). |
| 1. Use warming mattress, blankets or warmed intravenous fluids if needed, to prophylactically prevent hypothermia. |
| 1. Reduce vasopressors (if possible) while maintaining hemodynamic stability. |
| **Donor management: monitoring** |
| 1. Monitor the core body temperature.   Target temperature: between 35-37°C. |
| 1. ECG monitoring of heart rate.   Target heart rate between 60-100 beats per minute. |
| 1. Repeat a 12-lead ECG for a potential heart donor if there are subsequent changes in monitored complexes. |
| 1. Invasive arterial pressure monitoring.   Target mean arterial pressure: ≥ 60 mm Hg. |
| 1. Measure additional parameters with extended monitoring in case of a patient with hemodynamic instability, by using for instance a pulmonary artery catheter, PiCCO or oesophageal Doppler. |
| 1. Measure additional parameters with extended monitoring in case of a patient with hemodynamic instability, by using transthoracic or transoesophageal echocardiography.   Target ejection fraction: ≥ 50 %. |
| 1. Ensuring a recent chest X-ray examination for a potential lung and/or heart donor is available. |
| 1. Monitoring of ventilator parameters. |
| 1. Periodically re-assess cuff pressure to check if there is no cuff leak and if cuff pressure is between 20-30 cm H_2_O to avoid aspiration. |
| 1. Peripheral oxygen saturation monitoring (SaO_2_).   Target SaO_2_: > 95 %. |
| 1. Perform a blood gas analysis on a regular basis.   Target pH: 7.3-7.5.  Target arterial oxygen tension (PaO_2_): 80-100 mm Hg.  Target arterial carbon dioxide tension (PaCO_2_): 35-45 mm Hg. |
| 1. Send a bronchial secretion sample for microscopy and culture if secretions are present. |
| 1. Perform a bronchoscopy for diagnosis or therapy if clinically indicated. |
| 1. Estimate the effective intravascular volume and overall fluid status by chart review and clinical examination. |
| 1. Monitor hourly urine output, particularly looking for any suggestion of the onset of diabetes insipidus (polyuria).   Target urine output: 0.5-3 mL/kg/h. |
| 1. Measure blood electrolytes on a regular basis.   Target serum sodium: ≤ 155 mEq/L. |
| 1. Monitoring of glycemic status.   Target blood glucose: ≤ 180 mg/dL. |
| 1. Measure routine full blood counts to examine the need for transfusion of red blood cells if clinically indicated.   Target hemoglobin: > 7 g/dL. |
| 1. Ensuring coagulation screening or thromboelastography to target therapy if there is a clinically relevant bleeding. |
| **Donor management: cardiovascular management (hypotension**) |
| 1. Treat the systemic arterial hypertension related to “adrenergic storm” of severe degree (MAP > 120 mm Hg) and prolonged (> 30 to 60 minutes) with calcium entry blockers or short-acting cardioselective beta-blockers. |
| 1. Use isotonic crystalloids for intravascular volume replacement and use blood products and colloids (albumin) for specific circumstances. |
| 1. Avoid hydroxyethyl starch (HES) for intravascular volume replacement. |
| 1. Ensuring an appropriate prescription of vasoactive drugs when correction of the volume deficit fails to achieve the threshold hemodynamic goals. |
| **Donor management: cardiovascular management (bradycardia)** |
| 1. Treat bradycardia causing hemodynamic instability, with a short acting β-adrenergic agonist (epinephrine/dopamine/dobutamine/isoprenaline) or occasionally transvenous pacing. Don’t use atropine because bradycardia are the consequence of high-level vagal stimulation and exhibit a high degree of resistance to atropine. |
| **Donor management: cardiovascular management (tachycardia)** |
| 1. Treat tachycardia by following the established advanced cardiopulmonary life support guidelines. |
| **Donor management: respiratory management** |
| 1. Ensuring a lung protective ventilation is installed:  - Minimum FiO_2_ to obtain a PO_2_ between 80-100 mm Hg - Tidal volume (Vt): 6-8 mL/kg (ideal body weight) - Plateau pressure: < 30 cm H_2_O - PEEP (Positive End Expiratory Pressure): 8-10 cm H_2_O |
| 1. Maintain 30-45° head of bed elevation to avoid aspiration. |
| 1. Perform recruitment maneuvers and repeat when indicated. |
| 1. Perform intermittent nasopharyngeal suction. |
| 1. Perform intermittent tracheal suction, by preference using a closed circuit. |
| 1. Apply a prescription of oral hygiene every 6 hours. |
| **Donor management: renal and electrolyte management (oliguria < 0.5 mL/kg/h)** |
| 1. Treat hypovolemia, hypotension and cardiac dysfunction and consider diuretic only if needed. |
| **Donor management: renal and electrolyte management (polyuria > 3 mL/kg/h)** |
| 1. Review the medical history, urinary and blood sample to exclude secondary polyuria: osmotic (Mannitol, hyperglycemia), induced (diuretic) or adapted (fluid overload). |
| 1. Confirm diabetes insipidus: urine specific gravity below 1.005 g/mL or trend towards hypernatremia/hyperosmolarity. |
| 1. Treat diabetes insipidus with sufficient fluid volume replacement to compensate polyuria and anti-diuretic hormone replacement.  - Fluid volume replacement with monitoring of electrolytes and blood glucose levels. - Anti-diuretic hormone replacement with desmopressin as a first line medication. |
| **Donor management: renal and electrolyte management (electrolyte disturbances)** |
| 1. Treat electrolyte disturbances. |
| **Donor management: hormone substitution** |
| 1. Ensuring a prescription of hydrocortisone to reduce the cumulative dose and administration duration of vasopressors: hydrocortisone 50 mg + continuous infusion of 10 mg/h until the aortic clamping. |
| 1. Ensuring a prescription of methylprednisolone for a potential liver donor: 250 mg bolus + 100 mg/hour until recovery of organs. |
| 1. Consider thyroid replacement therapy for hemodynamically unstable donors or for potential heart donors with abnormal (<45%) left ventricular ejection fraction. |
| 1. Ensuring an appropriate prescription of insulin if treating hyperglycemia to achieve a target glucose level of 180 mg/dL or less. |
| **Post procurement care** |
| 1. Detection, registration and reporting of serious adverse events to the transplant center. |
| 1. Debriefing by the donor coordinator and/or transplant coordinator about the results of the transplantation (anonymous) to the relatives, health care professionals and primary care physician. |
| 1. Offering, if necessary, support to the relatives, for example by a feedback conversation after a couple of weeks or information about associations for relatives. |
| 1. Debriefing with the involved health care professionals and transplant coordinator. |
| 1. Ensuring the hospitalization invoice of the patient is excluded of any medical, pharmaceutical or hospital costs after the determination of brain death and legal declaration of death. |

**QUESTIONNAIRE PART III:**

**QUALITY INDICATORS (DELPHI ROUND 2)**

**First round**: please comment on the listed quality indicators if they are not well formulated for you or add new ones.

*For example: quality indicator X*

*Comments on this quality indicator: ……………………………………………………………..………..*

*Reference(s): ………….……………………………………………………………………………………….*

**Second & third round**: please evaluate the listed set of quality indicators for the attributes relevance and feasibility on a 9-point Likert Scale, with 1 indicating “strongly disagree” and 9 “strongly agree”.

- Relevance: the indicator truly measures the quality of care for the management of a donor after brain death in a perspective of organ donation (that is useful for professionals) [29].
- Feasibility: are data available and collectable, albeit contained within medical records or health authority datasets [29]?

*For example: quality indicator X*

|  | Strongly disagree |  |  |  | Undecided |  |  |  | Strongly agree |
| --- | --- | --- | --- | --- | --- | --- | --- | --- | --- |
|  | 1 | 2 | 3 | 4 | 5 | 6 | 7 | 8 | 9 |
| Relevance |  |  |  |  |  |  |  |  |  |
| Feasibility |  |  |  |  |  |  |  |  |  |

**Definition of consensus** **after the third round**

A quality indicator will be accepted with agreement if the attribute relevance has a median score of 7 or more with 75% of more of the ratings in the highest tertile (Likert score: 7-9) and the attribute feasibility has a median score of 7 or more.

| **Structure indicators**  *A measure that indicates the type and amount of resources used by an organization to deliver a key intervention.* |
| --- |
| 1. Existence of donation process procedures. |
| *Formula: existence of procedures for all relevant steps of the donation process?* |
| 1. Existence of a proactive donor detection protocol. |
| *Formula: existence of a donor detection protocol?* |
| 1. Donation team (see glossary) full-time availability. |
| *Formula: availability of the donation team 24/7?* |
| 1. Documentation of key interventions of the donation process. |
| *Formula: existence of a documentation form with all relevant key interventions of the donation process?* |
| 1. Seminars on organ donation. |
| *Formula: number of organ donation seminars organized last year?* |
| **Process indicators**  *A measure that indicates the performance of (compliance with) a key intervention.* |
| 1. Detection of all potential donors after brain death in the ICU. |
| *Formula: number of potential donors after brain death in the ICU who are referred to the donor coordinator / number of potential donors after brain death in the ICU.* |
| 1. Evaluation of donors after brain death. |
| *Formula: number of patients declared brain death in the ICU who have been evaluated as donors in consult with a transplant center / number of patients declared brain death in the ICU.* |
| 1. Donor management goals. |
| *Formula: number of actual donors after brain death (see glossary) in the ICU meeting 5 of the 7 donor management goals prior to organ recovery (mean arterial pressure: 60-110 mm Hg, number of vasopressors ≤ 1, arterial blood gas pH: 7.3-7.5, serum sodium: 135-155 mEq/L, blood glucose: ≤ 180 mg/dL, urine output: ≥ 0.5 mL/kg/h over 4 hours, core body temperature: 35-37°C) / number of actual donors after brain death in the ICU.* |
| 1. Documentation of cause of no donation. |
| *Formula: number of failed potential donors in which the cause of no donation is properly documented / number of failed potential donors.* |
| 1. Documentation of evaluation of potential donors. |
| *Formula: number of donors correctly evaluated / number of donors evaluated.* |
| **Outcome indicators**  *A measure that indicates the result of a performance (or non-performance) of a key intervention.* |
| 1. Family objection to organ donation. |
| *Formula: number of objections (number of potential donor after brain death cases with family objection to organ donation) / number of families interviewed* (number of potential donor after brain death cases in which family members are informed about the possibility of organ donation). *exclusion of donor cases where the patient’s wishes are known (formal or informal).* |
| 1. Conversion rate in donors after brain death. |
| *Formula: number of actual donors after brain death / number of eligible donors after brain death (see glossary).* |

**Glossary**

**Potential donor after brain death**: a person whose clinical condition is suspected to fulfill brain death criteria [30].

**Eligible donor after brain death**: a medically suitable person who has been declared death based on neurologic criteria as stipulated by the law of the relevant jurisdiction [30].

**Actual donor after brain death:**

A consented eligible donor [30]:

- 1. In whom an operative incision was made with the intent of organ recovery for the purpose of transplantation.

OR

- 1. From whom at least one organ was recovered for the purpose of transplantation.

**Utilized donor after brain death**: an actual donor from whom at least one organ was transplanted [30].

**Donation team:** the local donor coordination function should be performed by a multidisciplinary team (or donation team) consisting of at least one nurse and one specialist physician with a special professional title in intensive care (with 5 years’ experience on an intensive care or emergency unit).

**References**
