# Supplementary material for: Development of key interventions and quality indicators for the management of an adult potential donor after brain death: a RAND modified Delphi approach
Source: BMC Health Serv Res. 2018 Jul 24;18:580. doi: 10.1186/s12913-018-3386-1 (PMC6056930; doi:10.1186/s12913-018-3386-1)
Supplement: Supplementary file 2 — Additional results of the third Delphi round and the physical meeting. (DOC 113 kb) [file 12913_2018_3386_MOESM2_ESM.doc]

**Additional results of the third Delphi round and the physical meeting**

**Table 1.** Results of the 15 key interventions for which no consensus was reached by the overall panel (n = 18) after the third Delphi round.

|  | **Based on literature (L) or expert panel (E)** | **Median** | **Tertile 7-9 (%)** | **Tertile 7-9 (n)** | **Rating of contribution*** |
| --- | --- | --- | --- | --- | --- |
| **Donor management: general care** | | | | | |
| Continue enteral feeding until otherwise instructed by the transplant center. | L | 7 | 56% | 10 | 73% |
| Continue an appropriate prescription of deep venous thrombosis prophylaxis (low molecular weight heparin). | L | 8 | 72% | 13 | 81% |
| Ensuring a prescription of low-dose dopamine with a dose of (and not exceeding) 4 µg/kg/min until the aortic clamping and halve the dosage or terminate the infusion earlier when circulatory adverse effects occurred in association with the dopamine infusion, such as tachycardia (> 120 beats per min) or a marked increase in blood pressure (MAP > 110 mm Hg). | L | 5 | 39% | 7 | 59% |
| **Donor management: monitoring** | | | | | |
| Measure additional parameters with extended monitoring in case of a patient with hemodynamic instability, by using for instance a pulmonary artery catheter, PiCCO or oesophageal Doppler. | L | 6 | 39% | 7 | 72% |
| Measure additional parameters with extended monitoring in case of a patient with hemodynamic instability, by using transthoracic or transoesophageal echocardiography.  Target ejection fraction: ≥ 50 %. | L | 7 | 67% | 12 | 78% |
| Periodically re-assess cuff pressure to check if there is no cuff leak and if cuff pressure is between 20-30 cm H_2_O to avoid aspiration. | L | 8 | 67% | 12 | 83% |
| Monitoring of glycemic status.  Target blood glucose: ≤ 180 mg/dL. | L | 8 | 72% | 13 | 80% |
| Ensuring coagulation screening or thromboelastography to target therapy if there is a clinically relevant bleeding. | L | 8 | 67% | 12 | 81% |
| **Donor management: cardiovascular management (hypertension**) | | | | | |
| Treat the systemic arterial hypertension related to “adrenergic storm” of severe degree (MAP > 120 mm Hg) and prolonged (> 30 to 60 minutes) with calcium entry blockers or short-acting cardioselective beta-blockers. | L | 7 | 67% | 12 | 77% |
| **Donor management: cardiovascular management (hypotension)** | | | | | |
| Avoid hydroxyethyl starch (HES) for intravascular volume replacement. | L | 8 | 72% | 13 | 85% |
| **Donor management: respiratory management** | | | | | |
| Perform intermittent nasopharyngeal suction. | L | 8 | 67% | 12 | 85% |
| Perform intermittent tracheal suction, by preference using a closed circuit. | L | 8 | 67% | 12 | 81% |
| **Donor management: hormone substitution** | | | | | |
| Ensuring a prescription of hydrocortisone to reduce the cumulative dose and administration duration of vasopressors: hydrocortisone 50 mg + continuous infusion of 10 mg/h until the aortic clamping. | L | 5 | 17% | 3 | 60% |
| Ensuring a prescription of methylprednisolone for a potential liver donor: 250 mg bolus + 100 mg/hour until recovery of organs. | L | 5 | 33% | 6 | 61% |
| Consider thyroid replacement therapy for hemodynamically unstable donors or for potential heart donors with abnormal (<45%) left ventricular ejection fraction. | E | 6 | 39% | 7 | 66% |

*rating of contribution = ratio of “sum of ratings on the intervention given by participants” to “sum of ratings on the intervention if all respondents rated the interventions as ‘strongly agree’”.

**Table 2.** Results of the quality indicator for which no consensus was reached by the overall panel (n = 18) after the third Delphi round.

|  | **Attribute** | **Median** | **Tertile 7-9 (%)** | **Tertile 7-9 (n)** |
| --- | --- | --- | --- | --- |
| **Structure indicator** | | | | |
| Donation team full-time availability. | Relevance | 8 | 72% | 13 |
| *Formula: availability of the donation team 24/7?* | Feasibility | 7 | 61% | 11 |

**Table 3.** Results of the 4 key interventions for which consensus was reached by 9 experts after the physical meeting.

|  | **Based on literature (L) or expert panel (E)** | **Median** | **Tertile 7-9 (%)** | **Tertile 7-9 (n)** | **Rating of contribution*** |
| --- | --- | --- | --- | --- | --- |
| **Donor management: general care** | | | | | |
| Continue an appropriate prescription of deep venous thrombosis prophylaxis (low molecular weight heparin). | L | 8 | 78% | 7 | 85% |
| **Donor management: monitoring** | | | | | |
| Periodically re-assess cuff pressure to check if there is no cuff leak and if cuff pressure is between 20-30 cm H_2_O to avoid aspiration. | L | 9 | 78% | 7 | 84% |
| Monitoring of glycemic status.  Target blood glucose: ≤ 180 mg/dL. | L | 7 | 78% | 7 | 84% |
| Ensuring coagulation screening or thromboelastography to target therapy if there is a clinically relevant bleeding. | L | 8 | 78% | 7 | 84% |

*rating of contribution = ratio of “sum of ratings on the intervention given by participants” to “sum of ratings on the intervention if all respondents rated the interventions as ‘strongly agree’”.

**Table 4.** Results of the 11 key interventions for which no consensus was reached by 9 experts after the physical meeting.

|  | **Based on literature (L) or expert panel (E)** | **Median** | **Tertile 7-9 (%)** | **Tertile 7-9 (n)** | **Rating of contribution*** |
| --- | --- | --- | --- | --- | --- |
| **Donor management: general care** | | | | | |
| Continue enteral feeding until otherwise instructed by the transplant center. | L | 5 | 33% | 3 | 63% |
| Ensuring a prescription of low-dose dopamine with a dose of (and not exceeding) 4 µg/kg/min until the aortic clamping and halve the dosage or terminate the infusion earlier when circulatory adverse effects occurred in association with the dopamine infusion, such as tachycardia (> 120 beats per min) or a marked increase in blood pressure (MAP > 110 mm Hg). | L | 5 | 22% | 2 | 52% |
| **Donor management: monitoring** | | | | | |
| Measure additional parameters with extended monitoring in case of a patient with hemodynamic instability, by using for instance a pulmonary artery catheter, PiCCO or oesophageal Doppler. | L | 5 | 0% | 0 | 53% |
| Measure additional parameters with extended monitoring in case of a patient with hemodynamic instability, by using transthoracic or transoesophageal echocardiography.  Target ejection fraction: ≥ 50 %. | L | 7 | 67% | 6 | 77% |
| **Donor management: cardiovascular management (hypertension**) | | | | | |
| Treat the systemic arterial hypertension related to “adrenergic storm” of severe degree (MAP > 120 mm Hg) and prolonged (> 30 to 60 minutes) with calcium entry blockers or short-acting cardioselective beta-blockers. | L | 3 | 33% | 3 | 53% |
| **Donor management: cardiovascular management (hypotension)** | | | | | |
| Avoid hydroxyethyl starch (HES) for intravascular volume replacement. | L | 9 | 56% | 5 | 60% |
| **Donor management: respiratory management** | | | | | |
| Perform intermittent nasopharyngeal suction. | L | 9 | 56% | 5 | 73% |
| Perform intermittent tracheal suction, by preference using a closed circuit. | L | 7 | 56% | 5 | 67% |
| **Donor management: hormone substitution** | | | | | |
| Ensuring a prescription of hydrocortisone to reduce the cumulative dose and administration duration of vasopressors: hydrocortisone 50 mg + continuous infusion of 10 mg/h until the aortic clamping. | L | 5 | 0% | 0 | 40% |
| Ensuring a prescription of methylprednisolone for a potential liver donor: 250 mg bolus + 100 mg/hour until recovery of organs. | L | 3 | 22% | 2 | 43% |
| Consider thyroid replacement therapy for hemodynamically unstable donors or for potential heart donors with abnormal (<45%) left ventricular ejection fraction. | E | 2 | 22% | 2 | 42% |

*rating of contribution = ratio of “sum of ratings on the intervention given by participants” to “sum of ratings on the intervention if all respondents rated the interventions as ‘strongly agree’”.

**Table 5.** Results of the quality indicator for which no consensus was reached by 9 experts after the physical meeting.

|  | **Attribute** | **Median** | **Tertile 7-9 (%)** | **Tertile 7-9 (n)** |
| --- | --- | --- | --- | --- |
| **Structure indicator** | | | | |
| Donation team full-time availability. | Relevance | 8 | 78% | 7 |
| *Formula: availability of the donation team 24/7?* | Feasibility | 5 | 33% | 3 |
